# Automated peptide mapping and protein-topographical annotation of proteomics data

**DOI:** 10.1186/1471-2105-15-207

**Published:** 2014-06-19

**Authors:** Pavankumar Videm, Deepika Gunasekaran, Bernd Schröder, Bettina Mayer, Martin L Biniossek, Oliver Schilling

**Affiliations:** 1 Institute of Molecular Medicine and Cell Research, University of Freiburg, D-79104 Freiburg, Germany; 2 Present address: Bioinformatics Group, Department of Computer Science, University of Freiburg, Freiburg, Germany; 3 Biochemical Institute, Christian-Albrechts-University of Kiel, D-24118 Kiel, Germany; 4 BIOSS Centre for Biological Signaling Studies, University of Freiburg, D-79104 Freiburg, Germany; 5 Stefan Meier Strasse 17, D-79104 Freiburg, Germany

**Keywords:** Peptide mapping, Quantitative proteomics, Trans proteomic pipeline

## Abstract

**Background:**

In quantitative proteomics, peptide mapping is a valuable approach to combine positional quantitative information with topographical and domain information of proteins. Quantitative proteomic analysis of cell surface shedding is an exemplary application area of this approach.

**Results:**

We developed ImproViser ( http://www.improviser.uni-freiburg.de) for fully automated peptide mapping of quantitative proteomics data in the protXML data. The tool generates sortable and graphically annotated output, which can be easily shared with further users. As an exemplary application, we show its usage in the proteomic analysis of regulated intramembrane proteolysis.

**Conclusion:**

ImproViser is the first tool to enable automated peptide mapping of the widely-used protXML format.

## Background

Peptide mapping is increasingly recognized as a valuable tool in quantitative proteomics. It integrates quantitative information of individual, typically tryptic, peptides with topographical protein annotation such as individual domains. Manual peptide mapping has established that matrix metalloprotease (MMP)-2 proteolytically releases the chemokine fractalkine into the pericellular milieu [1]. Peptide mapping is also crucial for correct functional annotation, e.g. distinguishing collagen cleavage products with signaling function from the actual collagen protein with a predominantly structural role [2].

Signal-peptide-peptidase-like (SPPL) proteases SPPL2a and –b cleave transmembrane proteins within the lipid bilayer with a preference for transmembrane proteins in type 2 orientation [3]. The few annotated substrates of SPPL2a and -b include tumor necrosis factor [4,5], the Fas ligand [6] and the invariant chain (CD74) of the major histocompatibility class II complex [7-10]. Common features of SPPL2a/b substrates include a short cytoplasmic tail and a large ectodomain. SPPL proteases release the cytoplasmic tail after initial shedding of the ectodomain by other proteases. From a proteomic perspective, quantitative alterations of the cytoplasmic tail are typically overshadowed by peptides stemming from the ectodomain. This makes peptide mapping useful in the proteomic analysis of SPPL proteolysis.

Some proteomic applications already include peptide mapping. A strategy termed PROTOMAP combines high coverage peptide mapping with size shift analysis to detect proteolytic truncation of proteins [11]. A novel tool termed QARIP [12] works with the proteomic software Maxquant [13] to analyze cell surface shedding by automated peptide mapping. Similarly, the PROTTER tool integrates experimental proteomics data with protein sequence annotations [14].

protXML is a well-established format to report protein identification and quantitation based on liquid chromatography–tandem mass spectrometry (LC–MS/MS). protXML is most prominently implemented by the Trans Proteomic Pipeline (TPP) [15], a set of open–source tools for quantitative proteomic data analysis. A large user community extensively employs the TPP which is known for supporting a large range of data formats and mass spectrometers [16].

Since the TPP continues to be a widespread tool, we aimed to develop a web–based service for fully automated peptide mapping analysis from quantitative protXML data together with protein topographical annotation. Our aims included:

1. Deconvolute global protein ratios by spatially resolving the underlying peptide ratios.

2. Facilitate the analysis of cell surface shedding by distinguishing extra- and intracellular ratios for membrane–spanning proteins.

3. Facilitate interpretation of proteomic results by linking protein IDs to the corresponding UniProt information [17].

4. Share these results with non–expert users and collaborators in a straightforward manner.

To fulfill these requirements, we developed ImproViser (Improved Visualizer of protXML data), which is freely accessible at http://www.improviser.uni-freiburg.de.

## Implementation

**Figure 1:**
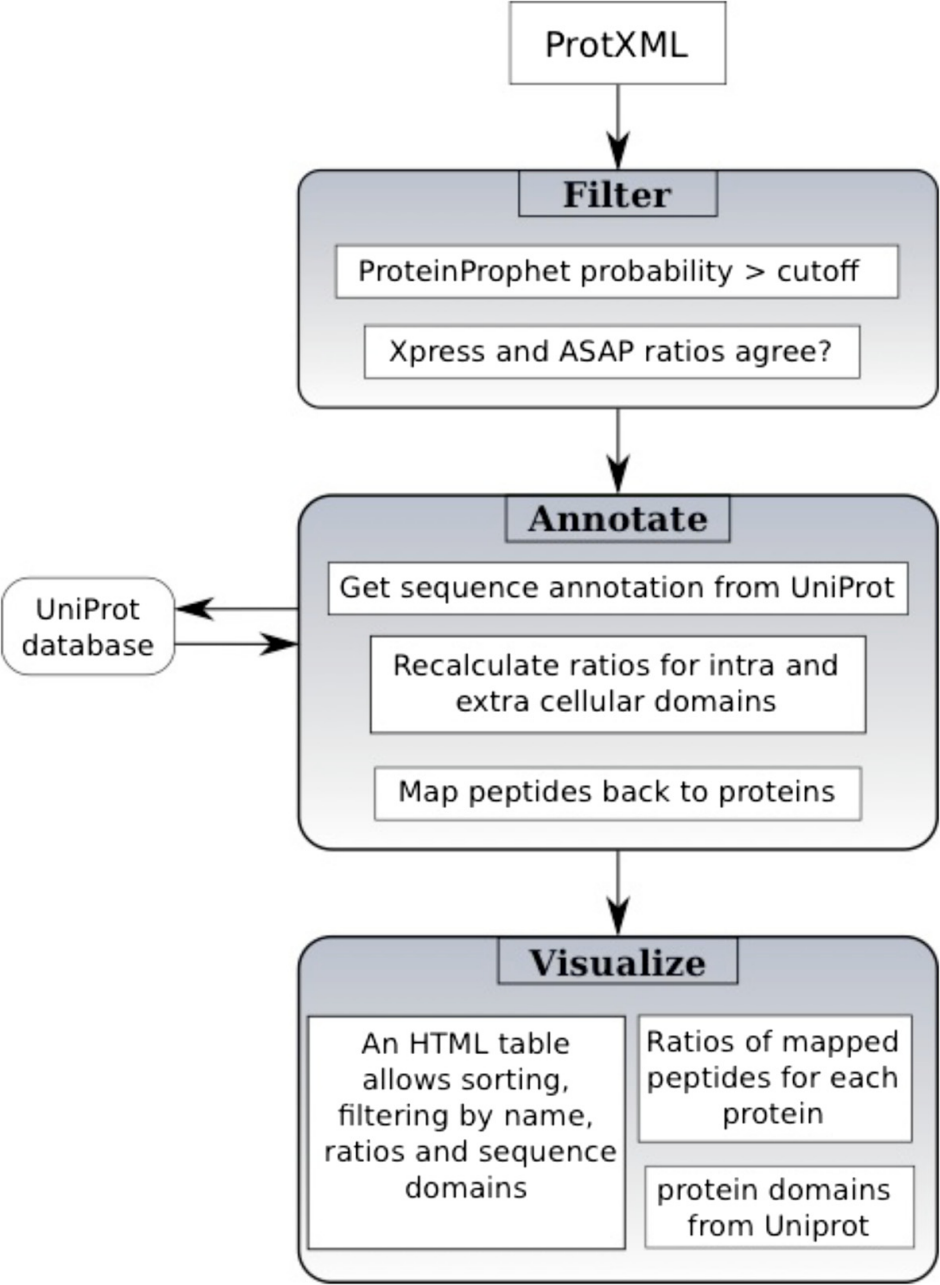
**Flowchart depicting the working procedure of the ImproViser tool.** The tool accepts a protXML file as input. Using information retrieved from this file, it refers to the UniProt database to obtain selected information and delivers it back to the ImproViser script, which generates a graphical table with all relevant information. This output is then compressed and can be downloaded as a zip file by the user.

ImproViser is a web-based platform implemented in Perl. It reads user–provided protXML data, which has been generated by ProteinProphet [18] as part of the TPP package [15]. ImproViser interacts with the UniProt database to obtain protein annotation. A compressed output file is generated for local storage and sharing. ImproViser output can be visualized using web-browsers with CSS3 support., i.e. *Firefox*. The workflow is shown in Figure 1.

ImproViser supports protein identifications originating from sequence databases that adhere to UniProt [17] or International Protein Index (IPI) nomenclature [19]. The tool obtains UniProt identifiers from the protXML file and subsequently retrieves the following entry-specific information from the UniProt database: recommended name; molecular weight; length; topological information such as presence and location of transmembrane regions, N-terminal signal peptides, cytoplasmic, and extra-cytoplasmic (e.g. extracellular, and luminal) domains.

By default, ImproViser rejects protein entries with a ProteinProphet probability score < 0.90 [18]. This value is user - adjustable. ImproViser further extracts the relative quantitation as represented by the “light to heavy (L:H) ratio” derived by stable isotope labeling in a typical quantitative proteomics experiment. The TPP includes two software tools for relative quantitation: ASAPRatio [20] and XPRESS [21], with ASAPRatio being a more advanced approach for protein and peptide quantitation. ImproViser reports ASAPRatio. The tool offers the possibility to validate ASAPRatio quantitation by XPRESS quantitation if such values are included in the protXML file. Such a validation strategy has been of use in some recent quantitative proteomic studies [2,22], especially since ASAPratio occasionally displays inaccuracies with regard to background removal and separation of neighboring peaks [23]. L:H ratios are log_2_ transformed. Further input options are described in Table 1.

**Table 1:** User-adjustable options

**Option**	**Variable name**	**Default value**	**Description**
Invert H and L	-insert	off	This function enables the user to invert the light to heavy ratios in the ProtXML file to heavy to light ratios.
Validate ASAPratio with Xpress	-validate	off	This function validates the ASAPRatio [8] (Automated Statistical Analysis on Protein Ratio) results with XPRESS [9] results.
Elaborate list of peptides	-elaborate	off	Selecting this function displays the list of all occurrences of a peptide (in case they are identified more than once, by default the tool chooses the peptide with highest Peptide Prophet probability score).
ProteinProphet cutoff	-threshold	0.90	This function enables the user to set the cutoff for the ProteinProphet probability score. Any protein with a score less than this cutoff is discarded.
Minimum peptide ratio	-rmin	-3.0	This function enables the user to set the minimum value allowed for light:heavy ratio of the peptide. This measure is then used for scaling of the peptide ratios.
Maximum peptide ratio	-rmax	3.0	This function enables the user to set the maximum value allowed for light:heavy ratio of the peptide. This measure is then used for scaling of the peptide ratios.
Negative no change zone	-zn	-0.25	This function enables the user to set the negative threshold for light to heavy ratio of the peptide. i.e. the peptide ratios between the Zn and Zp thresholds are categorized together.
Positive no change zone	-zp	0.25	This function enables the user to set the positive threshold for light to heavy ratio of the peptide. i.e. the peptide ratios between the Zn and Zp thresholds are categorized together.

Protein entries are considered as being “valid” if they pass the criteria described above. For each valid protein entry, ImproViser retrieves annotation from UniProt as described above. In addition, the tool extracts the individual peptide L:H ratios (as determined by ASAPRatio) for each valid protein entry. Peptide ratios are normalized as described above, log_2_ transformed, and graphically mapped on the linear protein sequence using a red - green scale to visualize individual peptide ratios. For protein regions that are explicitly annotated as being cytoplasmic or extra-cytoplasmic, ImproViser calculates a novel average ratio.

ImproViser is accessible via http://www.improviser.uni-freiburg.de. A test data set is also available for download. The user uploads an input protXML file and the tool generates an output HTML file (named index.html), which enables a tabulated visualization of the input. The tool outlines the details of the identified proteins and peptides. It also enables the user to select proteins based on specific features such as presence of N-terminal signal peptides and presence of transmembrane regions. The tool further generates (a) a log file which contains a list of proteins that were discarded (named run_stats.out), (b) a .txt file containing the information about the average molecular weight of the proteins listed in the output HTML file (named average_molecular_weight.txt), (c) a .txt file describing the system requirements and browser compatibility for viewing the output HTML file in its intended format (named suppoted_browsers_and_os.txt), (d) folders for storing images which are displayed in the index.html file (named images, small_images), and (e) a folder for storing HTML file link for specific proteins (named index_files). ImproViser also copies the necessary java scripts and css files required for the script to generate the formatted output. The formatted output produced by the tool is supported by all css3 compatible web browsers. The above-mentioned files are compressed in a zip format and presented for download. In our experience, the file size is often below 10 MB, thus allowing for easy sharing with collaborators via e-mail or file transfer services.

## Results and discussion

### General

**Figure 2:**
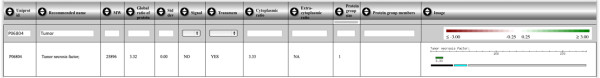
Screenshot of output HTML file (index.html) generated by ImproViser using a the data set reported in this manuscript.

A screenshot of an exemplary analysis is depicted in Figure 2. The different analysis options are described in Table 1. The performance of the ImproViser tool was investigated using the test data set. The size of the ProtXML test data was approximately 2.5 Mb (containing 625 proteins). The time taken for output generation was approximately 4 min. The size of the compressed output file was approximately 5 Mb.

### Application to proteomic analysis of SPPL - mediated intramembrane proteolysis

As outlined above, SPPL2a and SPPL2b typically cleave type 2 transmembrane proteins with short cytoplasmic tails following the initial proteolytic shedding of a larger ectodomain. For proteomic analysis of putative SPPL2a/b substrates, bone marrow derived dendritic cells (BMDCs) were prepared from mice deficient for both SPPL2a and SPPL2b (*SPPL2a*^*-/-*^*SPPL2b*^*-/-*^). Control BMDCs were generated from bone marrow of wild-type mice. BMDC isolation and culture has been performed as described previously [7]. Subsequently, cells were harvested and mechanically disrupted. Total cellular membranes were recovered by ultracentrifugation from a post-nuclear supernatant and washed with 100 mM sodium carbonate, pH 11.5, in order to enrich integral membrane proteins as described previously [24]. Following tryptic digestion in the presence of the acid labile surfactant RapiGest (Waters), peptides were dimethylated with stable isotopic forms of formaldehyde as described previously [2,22]. LC-MS/MS and corresponding data analysis with the TPP were also performed as described previously [2,22]. The resulting prot.xml file was further analyzed by ImproViser.

**Figure 3:**
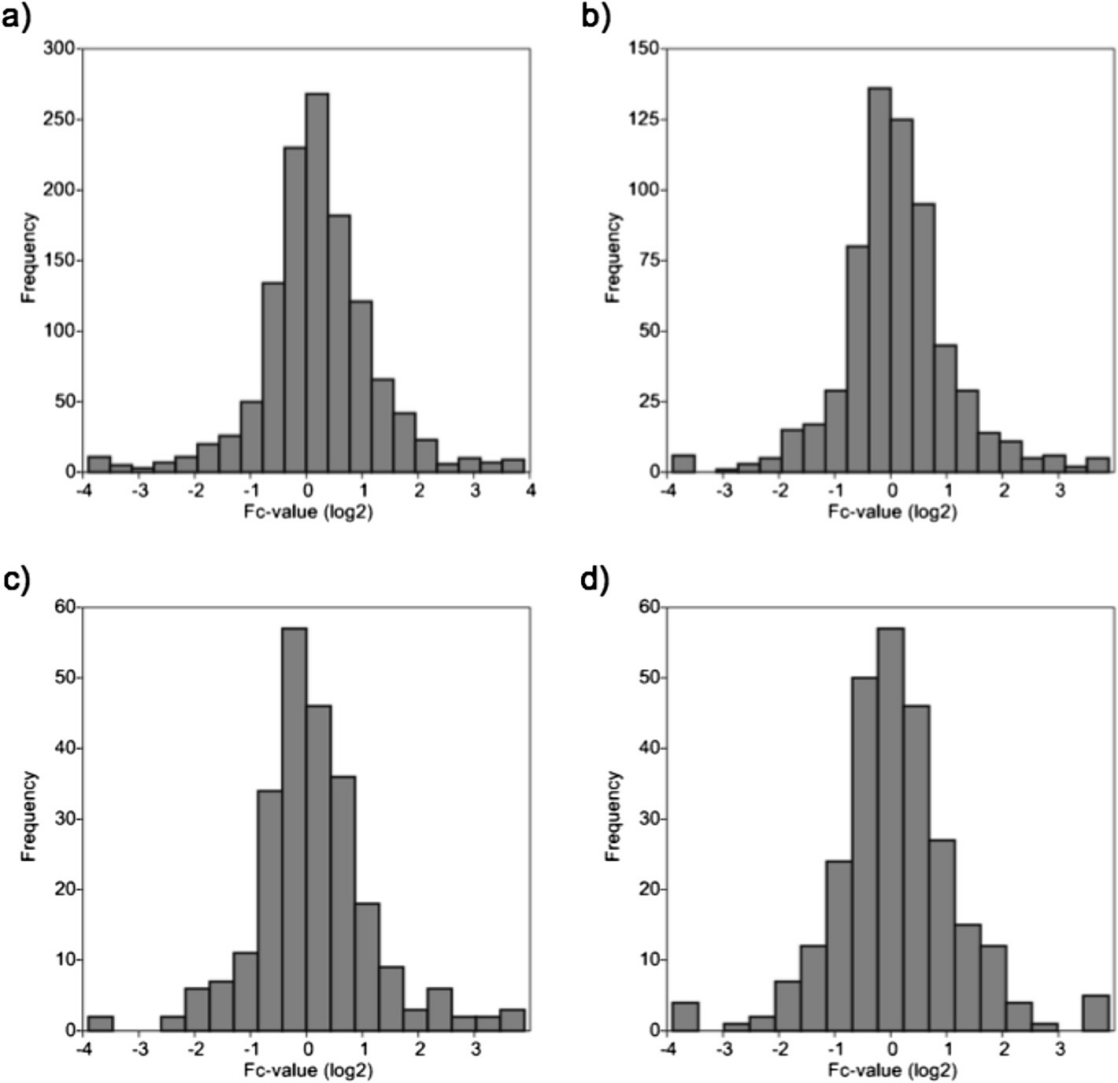
**Distribution of fold-change values (log**_**2 **_**of light: heavy ratios) for the proteomic analysis of a murine BMDC membrane fraction comparing wild-type cells to *SPPL2a***^***-/- ***^***SPPL2b***^***-/- ***^**cells. (a)** all identified proteins, **(b)** membrane-spanning proteins, **(c)** cytoplasmic domains of membrane-spanning proteins, **(d)** non-cytoplasmic domains of membrane-spanning proteins (e.g. extracellular, lumenal).

**Figure 4:**
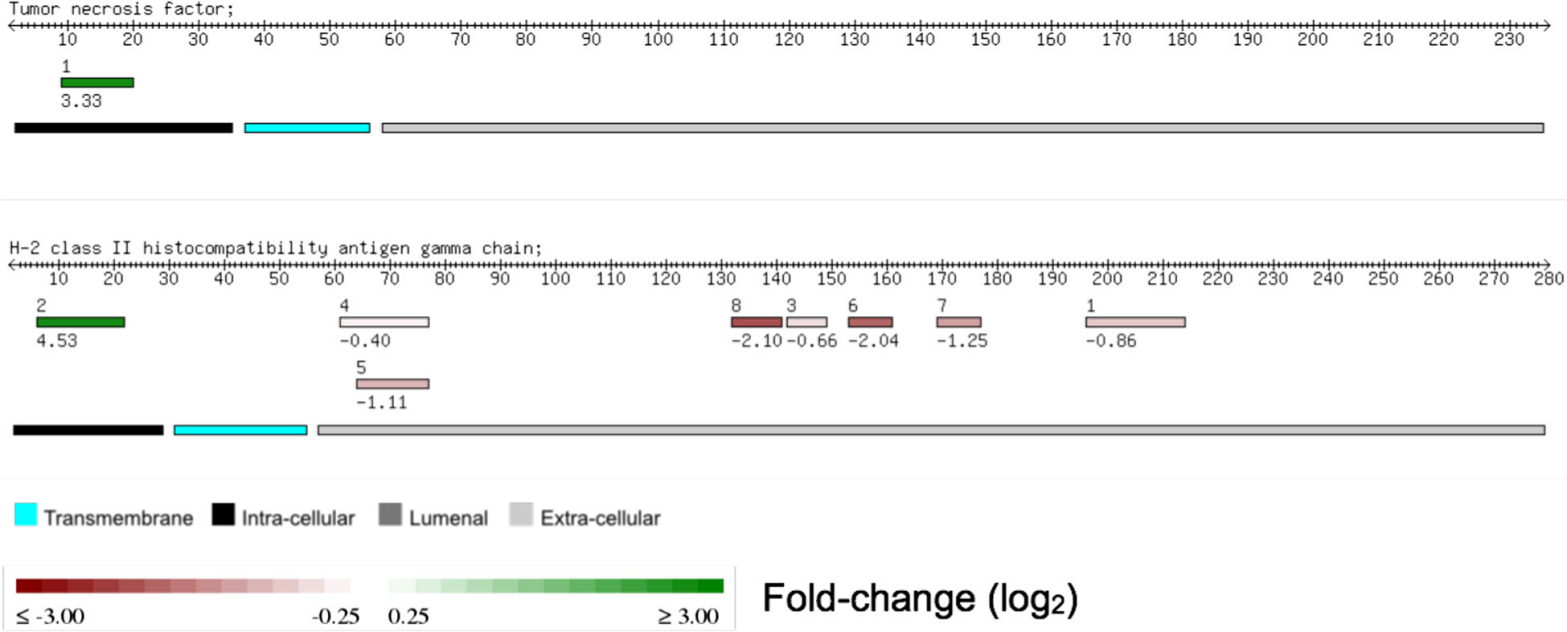
**Peptide mapping for known substrates of SPPL2a and -2b, as determined by the proteomic analysis of a murine BMDC membrane fraction comparing wild-type cells to *SPPL2a***^***-/-***^ ***SPPL2b***^***-/- ***^**cells.**

The proteomic analysis of the membrane-enriched fraction from wild-type and *SPPL2a*^*-/-*^*SPPL2b*^*-/-*^ BMDCs identified and quantified a total of 1231 proteins (Table 2). Of these, 629 (51%) featured a transmembrane domain as annotated by Uniprot. The large proportion of membrane spanning proteins indicates successful enrichment membrane proteins from the BMDCs. To a large extent (73%), Uniprot did not report a signal peptide sequence for the membrane spanning proteins.Overall, the fold-change (Fc) values (log2 of light to heavy ratios) for all identified proteins followed a near normal distribution with most proteins displaying none or very little quantitative alteration (Figure 3a). A similar near-normal Fc-value distribution is also observed for membrane-spanning proteins with regard to their global Fc-values (Figure 3b) as well as Fc-values based on peptides stemming from cytoplasmic or extra-cytoplasmic protein regions (Figure 3c,d). These data underline that deletion of SPPL2a and -2b does not result in major perturbations of the BMDC membrane proteome.We hypothesized that lack of SPPL2a and -2b activity leads to an accumulation of N-terminal fragments of putative SPPL substrates in the BMDC membrane fraction, as compared to the corresponding wild-type cells. Based on our knowledge of currently recognized SPPL2a/b substrates (see Introduction), further criteria for SPPL substrates are classification as a type 2 transmembrane protein with a single membrane spanning domain together with the presence of an extended ectodomain and a short cytoplasmic tail. To identify putative substrates of SPPL2a and -2b, we focused on proteins that adhere to this topology. Further selection criteria were a more than 2-fold accumulation (Fc-value > 1) of the cytoplasmic tail. Three proteins match these criteria: tumor necrosis factor, invariant chain (CD74) of the major histocompatibility class II complex, and macrophage receptor MARCO. Of these, tumor necrosis factor, invariant chain (CD74) of the major histocompatibility class II complex represent established SPPL substrates, thus validating our approach (Figure 4).For the invariant chain (CD74) of the major histocompatibility class II complex, opposing quantitative alterations are found for the cytoplasmic tail and ectodomain: while the short cytoplasmic tail accumulates, the longer ectodomain shows decreased levels (Figure 4). Due to its larger size, multiple peptides stem from the ectodomain as opposed to a single cytoplasmic peptide which spans most of the cytoplasmic tail. Without accurate protein topological annotation, accumulation of the cytoplasmic tail would be overshadowed by more numerous ectodomain peptides with opposing Fc-values. This example further illustrates the usefulness of peptide mapping in functional annotation of quantitative proteomics data.

**Table 2:** Proteomic analysis of the murine BMDC membrane fraction

Total proteins identified and quantified	1231
– with annotated transmembrane domain	629
– with annotated signal peptide sequence and with signal peptide sequence	171
– with annotated transmembrane domain but without signal peptide sequence	458
– with annotated transmembrane domain and quantified peptides of cytoplasmic localization	385
– with annotated transmembrane domain and quantified peptides of extra-cytoplasmic localization	362

It is an intrinsic feature of every peptide mapping approach that quantitations of protein domains are based on less peptide features than those for the entire protein. For example, the cytoplasmic tail of the invariant chain (CD74) of the major histocompatibility class II complex encompasses 29 amino acids with one tryptic peptide of 17 residues. The reduced number of peptide features employed in domain quantitation necessitates particular care in the interpretation of such results since individual peptide quantitations are prone to poor chromatographic resolution [23] or non-dynamic behaviour in quantitative proteomic analysis [25].

## Conclusion

Peptide mapping is a useful additional level of proteomic data analysis. The ImproViser tool serves as a platform to automate this process and provides a graphical representation of protXML data, as highlighted by an exemplary proteomic analysis of regulated intramembrane proteolysis. We consider quantitative proteomic analysis of cell surface shedding to be a major application area of ImproViser. It might also be of interest for the proteomic analysis of other post-translational modifications such as phosphorylation.

## Availability and requirements

**Project name:** ImproViser

**Project home page:**http://www.improviser.uni-freiburg.de

**Operating system:** Platform independent

**Programming language:** Perl

**Other requirements:** Requires web browsers that support css3, hence recent versions of Firefox, Chrome and Opera are recommended.

**License:** ImproViser is available freely online at http://www.improviser.uni-freiburg.de

**Any restrictions to use by non-academics:** none

## Competing interest

The authors declare no conflict of interest.

## Authors’ contributions

DG and PV programmed ImproViser. BM and BS prepared the samples. MB performed the mass spectrometric analysis. OS designed the concept and supervised the work. All authors participated in preparing the manuscript. All authors read and approved the final manuscript.
